# FitKids360: Design, Conduct, and Outcomes of a Stage 2 Pediatric Obesity Program

**DOI:** 10.1155/2014/370403

**Published:** 2014-08-20

**Authors:** Jared M. Tucker, Joey C. Eisenmann, Kathleen Howard, Emily H. Guseman, Kimbo E. Yee, Kimberly DeLaFuente, Jill Graybill, Meggie Roberts, Megan Murphy, Heather Saturley, Tom Peterson

**Affiliations:** ^1^Helen DeVos Children's Hospital, Grand Rapids, MI 49503, USA; ^2^Division of Sports & Cardiovascular Nutrition, Michigan State University, East Lansing, MI 48824, USA; ^3^Forest Hill Pediatrics, Grand Rapids, MI 49546, USA; ^4^Division of Kinesiology & Health, University of Wyoming, Laramie, WY 82071, USA; ^5^Department of Kinesiology, Michigan State University, East Lansing, MI 48824, USA; ^6^Spectrum Health Healthier Communities, Grand Rapids, MI 49504, USA; ^7^College of Human Medicine, Michigan State University, East Lansing, MI 48825, USA; ^8^Grand Valley State University, Allendale, MI 49401, USA; ^9^Health Net of West Michigan, Grand Rapids, MI 49503, USA; ^10^Sisters of Charity of Leavenworth Health System, Denver, CO 80021, USA

## Abstract

This paper describes FitKids360, a stage 2 pediatric weight management program. FitKids360 is a physician-referred, multicomponent, low-cost healthy lifestyle program for overweight and obese youth 5–16 years of age and their families. FitKids360 provides an evidence-based approach to the treatment of pediatric overweight by targeting patients' physical activity, screen time, and dietary behaviors using a family-centered approach. The intervention begins with a two-hour orientation and assessment period followed by six weekly sessions. Assessments include lifestyle behaviors, anthropometry, and the Family Nutrition and Physical Activity (FNPA) survey, which screens for obesogenic risk factors in the home environment. Outcomes are presented from 258 patients who completed one of 33 FitKids360 classes. After completing FitKids360, patients increased moderate to vigorous physical activity by 14 minutes (*P* = 0.019), reduced screen time by 44 minutes (*P* < 0.001), and improved key dietary behaviors. Overall, FNPA scores increased by 9% (*P* < 0.001) and 69% of patients with “high risk” FNPA scores at baseline dropped below the “high risk” range by followup. Patients also lowered BMIs (*P* = 0.011) and age- and sex-adjusted BMI *z*-scores (*P* < 0.001) after completing the 7-week program. We hope this report will be useful to medical and public health professionals seeking to develop stage 2 pediatric obesity programs.

## 1. Introduction

The epidemiologic evidence of the prevalence and consequences of pediatric obesity is well documented [1]. Currently, there is considerable emphasis on establishing effective prevention and treatment protocols for pediatric obesity [2, 3]. In 2007, an expert committee put forth recommendations for the assessment, prevention, and treatment of pediatric obesity [2]. In this report, a staged approach to the prevention and treatment under the chronic care model was outlined. In brief, four treatment stages consisting of increasing intensity were recommended. Patients generally begin at the least-intensive stage and advance depending on responses to treatment, age, severity of obesity, health risks, and motivation.

Stage 1 is referred to as “prevention plus” and generally takes place in the primary care office setting. Key lifestyle behaviors are promoted and if, after 3 to 6 months, the child has not made appropriate improvement the provider can offer the next level of obesity care. Stage 2 entails structured weight management and often includes a multidisciplinary team of health care providers. This level of obesity treatment is distinguished from prevention plus less by differences in the targeted health behaviors and more by the support and structure that is provided to the child in achieving health behaviors. Stages 3 and 4 occur within specialty clinics and include a multidisciplinary team where the intensity of behavior changes and frequency of visits increases. In stage 4 obesity care, medications, very low calorie diets, or bariatric surgery are prescribed.

This paper describes the methodology of FitKids360, a stage 2 pediatric weight management program, and provides outcomes in patients who have completed one of 33 FitKids360 classes conducted between 2010 and 2013.

## 2. What Is FitKids360?

### 2.1. Overview

FitKids360 is a multicomponent, family-based, low-cost healthy lifestyle program targeting overweight and obese children and adolescents of 5–16 years of age and their families. FitKids360 was conceived and designed by a multidisciplinary team of medical professionals (pediatricians, nurses, dieticians, social workers, exercise specialists, or leaders). The overall goal of FitKids360 is to provide an evidence-based approach to the treatment of pediatric overweight and obesity by achieving the following primary aims: (1) improving the patients' physical activity, screen time, and dietary behaviors and (2) improving the family's “obesogenic” risk score.

The “360” portion of the program name was suggested by the marketing agency that designed the FitKids360 material. It stems from the logo being 360 degrees and is intended to depict people coming full circle, being well rounded, and the program being part of everything in their life. In addition, it was to represent that the program was of full service to encompass all aspects of obesity, not just the physical component, and to support our belief that a 360-degree perspective is required to address the complex issue of pediatric obesity.

The involvement of the family and assisting participants by creating a supportive environment for healthy behaviors is a major component of FitKids360. Indeed, the family component is essential in the development of an environment that supports children's efforts to implement changes in nutrition, physical activity, and screen time [4, 5]. Furthermore, parents and other family members provide the primary social learning environment in which attitudes and behaviors regarding eating, physical activity, and the use of screen media are formed. The strong influence of the relationship between the parent/caregiver and child, including modeling of health behaviors, creating an environment conducive to active lifestyles, choosing and preparing food, and encouraging and reinforcing eating and physical activity patterns, suggests that parents and caregivers must be involved in interventions designed to increase healthy eating and physical activity in childhood. Epstein and Wing [6] cited 3 reasons for parental and familial involvement in obesity interventions. (1) Because obesity runs in families, it may be unrealistic to intervene with one family member while other family members are modeling and supporting behaviors that may counteract the intervention's effectiveness; (2) specific parental behaviors that facilitate overeating and inactivity are important in the development of unhealthy behaviors; (3) to achieve maximal behavior change in children, use of specific behavior-change strategies (such as positive reinforcement) by parents may be warranted.

### 2.2. Historical Background/Development of FitKids360

For background purposes, it is important to note that this program was developed based on a collaborative design; there was no grant funding or hired staff. It was a true community collaborative at its best. Beginning in 2009, key players were actively recruited, including researchers, marketing experts, nonprofits, insurance company representatives, fitness centers, and primary care physicians. As the concept was designed, volunteers filled the needs in the constructing of the curriculum, program development, brand design, and so forth. The original objectives of the program were to be (1) evidence-based, (2) free of charge, and (3) widely available at multiple sites and times within the community (up to 20 or more sessions per year).

### 2.3. Patient Population and Referral Process

Patients must be 5 to 16 years of age and have a body mass index (BMI) ≥ 85th percentile (i.e., overweight or obese) according to the Centers for Disease Control growth chart [7]. In addition, since FitKids360 utilizes a family-based approach, a parent or guardian must accompany the patient to each class, including the orientation session, and is expected to be involved and assist in making positive changes and offer vital encouragement throughout the duration of the program. Siblings are also encouraged to attend. Each session is limited to 20–25 children and their parents in order to give individual attention to each family and build relationships with them. To date, we have targeted low-income, underserved youth and their families with approximately 85% of participants enrolled in Medicaid.

All patients must be referred by a physician or health care provider to the FitKids360 program coordinator. Self-referrals must go through a physician or health care provider. In addition, patients and their parents or support partners are screened for readiness to change [8]. The program coordinator enters the patient's information into a wait list and, upon announcement of a new class, places a phone call to the patient (descending order on the referral list). Following three attempts to contact the patient, the program coordinator will refer the patient back to the referring physician. Patients who are contacted and ready to initiate the program are mailed a welcome letter and also receive a reminder phone call the day before the class is scheduled to start.

### 2.4. FitKids360 Staff

FitKids360 is conducted by a multidisciplinary team of experts in their respective discipline and in pediatric obesity.  Table 1 provides a description of the role and responsibilities for each staff member involved in FitKids360.

### 2.5. The FitKids360 Intervention

The intervention begins with a two-hour orientation and assessment period followed by six weekly sessions. Each weekly session is two hours in duration and consists of physical activity and nutrition education and behavioral counseling for the patients and their social support person(s). In addition, physical activity is performed intermittently throughout the session for a total of 30–60 minutes. Between weekly sessions, the patients log habitual physical activity, screen time, and dietary behaviors.

#### 2.5.1. Orientation

The orientation consists of an introduction of the FitKids360 staff, a description of the purpose of the program, program expectations, class structure, and a review of the weekly log sheets and other paperworks. In addition, preintervention assessments are conducted (see below for details).

Besides general introductions of all participants, we also conduct “getting to know you/icebreaker” activitiesto create a connection to the group. For example, if your name were given as the description for any one word in the dictionary, what word would that be? And why? We also try to provide a positive perception of pediatric obesity treatment by starting the discussion with the following questions.

- What do you think this group will be about and why do you think you were asked to participate?
- What are your perceptions of the group? What things do you think you have in common?
- What needs to happen for your family to be successful?

#### 2.5.2. Curriculum

The curriculum was developed based on an evidence-based approach to key features related to pediatric obesity. “Healthy counts” (8-7-6-5-4-3-2-1-0) are the cornerstone of the FitKids360 curriculum and more broadly our primary care and public health education campaign:

- 8 to 11 hours of sleep every night;
- 7 breakfasts every week;
- 6 home-cooked meals around the table every week;
- 5 servings of fruit and vegetables every day;
- 4 positive self-messages per day;
- 3 servings of low-fat dairy per day;
- 2 hours or less of screen time per day;
- 1 hour or more of physical activity per day;
- 0 sugar-sweetened beverages per day.

More specifically, the cornerstone lifestyle behaviors of physical activity/sedentary behavior and nutrition were coupled with behavioral strategies and issues. The FitKids360 development team relied upon the expert committee recommendations [2] to develop the curriculum, which covered various behavioral, nutritional, and physical activity topics across the duration of the program. The outline of this curriculum is described in Table 2.

#### 2.5.3. Tracking Logs and Incentive Program

Families monitor their progress towards their goals via tracking logs for diet (grains, vegetables, fruits, dairy, meat, and beans), physical activity, and screen time. Patients and their families set weekly goals with support from the clinical staff. Besides monitoring goals, the tracking logs are also part of the incentive point system (Table 3). Weekly prizes (e.g., hacky sacks, inflatable beach balls, water bottles, jump ropes, hula hoops, and other fun, active prizes) are offered to incentivize attending each class and participating in all activities. During the last class session, grand prizes are awarded based on incentive points earned.

#### 2.5.4. Attendance and Compliance

To date, 258 of 418 (62%, 38% attrition rate) patients who started the program have completed it. In comparison, a recent review reported attrition rates ranging from 27% to 73% (with most on higher end) [9]. Aside from individual patient and family motivation, we have implemented a few procedures that probably contribute to this low attrition rate. First, the program or site coordinator makes weekly phone calls 24–48 hours before class to remind parents about the class and confirm attendance. Second, transportation via taxi is provided free of charge to patients if they are not able to get to class on their own. Third, childcare is provided for children under the age of five years. Parents are encouraged to bring siblings over the age of 5 years to the class.

### 2.6. The Buddy Program

While the weekly curriculum provides patients and their families with the foundation for a healthy lifestyle, the Buddy Program presents the opportunity for enhanced and extended support especially since compliance and adherence are major barriers in the treatment of childhood obesity. The Buddy Program serves as a transition program that helps children and their families translate knowledge from weekly sessions into lasting behavior changes.

A “Buddy” is a case manager and mentor. The mentoring approach used here is similar to that used in public schools for a variety of goals (e.g., educational, life skills, and health habits) [10, 11]. A recent school-based intervention [12] has also incorporated mentoring by trained college allied health and medical students using adapted facets of goal setting methodology previously used with adults [13, 14], with insights and guidance from the limited published research on individualized goal setting for health behavior change with youth [15, 16].

The first phase of the Buddy Program, which occurs concurrently with the FitKids360 weekly sessions, matches participating families with student mentors (Buddies) for accountability, goal setting, motivation, and weekly planning. The Buddies are first and second year students at the Michigan State University College of Human Medicine. Each medical student is assigned one or two families during the first week of class. The Buddies engage patients and their families in weekly communication via phone calls. The minimum requirements for each contact include (1) asking about progress towards individual and family goals, (2) remembering to complete physical activity and screen time logs, and (3) addressing any remaining concerns.

The second phase of the Buddy Program occurs during the 6 months following completion of the FitKids360 weekly sessions. This phase builds on the relationships formed during the FitKids360 class session and includes continuation of phone calls or email “checkups” to facilitate structured goal setting and monthly family events to review themes from the FitKids360 classes and demonstrate healthy lifestyle choices (e.g., healthy cooking demonstrations).

This opportunity for medical students to engage in a pediatric obesity treatment program is unique, since the medical school curriculum does not typically cover details of nutrition, physical activity, healthy lifestyle, or obesity prevention and treatment. This is important since residency and physician training after residency graduation consist of individuals choosing courses on their own to meet continuing medical education requirements without mandatory update training in any specific areas of medicine. Furthermore, training of future physicians in obesity prevention and treatment is critical regardless of their future career paths. Whether they choose primary care or subspecialty paths, obesity does not spare any particular underlying disease process and its presence will complicate the disease processes of children with special health care needs.

## 3. Assessments

### 3.1. Measurement of Primary and Secondary Endpoints

Assessments are conducted at the beginning and conclusion of the program. The assessment team has consisted of site staff, nurses, medical students, and undergraduate and graduate health science students. These individuals are trained by one of the principal investigators (JMT). The assessment battery includes lifestyle behaviors (physical activity, screen time, and diet) as well as physical measures (height, weight, waist circumference, and percent body fat).

### 3.2. Primary Outcomes

#### 3.2.1. Lifestyle Survey

Physical activity, screen time (viewing television, playing video games, and online computer use), and diet are determined by self-report. The self-report question for physical activity is the same question used in the Youth Risk Behavior Survey, asking how many days per week children are engaged in moderate-to-vigorous physical activity (MVPA). Total screen time is determined from self-reported television viewing, computer use, and video games on weekdays and weekends. Dietary consumption of 100% fruit juices, fruit, vegetables, whole grains, sugar-sweetened beverages, sweets/desserts, and dairy was reported as times consumed per day or week.

#### 3.2.2. Family Nutrition and Physical Activity Score

Since the family environment is instrumental to the physical activity, screen time, and nutritional behaviors of the patient, the impact of the program on the family “obesogenic” environment is evaluated using the Family Nutrition and Physical Activity (FNPA) screening tool. The FNPA screening tool was developed by Ihmels and colleagues [17] through comprehensive evidence analyses supported by the American Dietetic Association and designed to determine the strength of evidence linking physical activity and diet behaviors with overweight/obesity in children. The evidence analyses identified ten primary factors (breakfast and family meals, nutrition modeling, nutrient dense foods, high calorie beverages, restriction and reward, parent modeling physical activity, child's physical activity, screen time, TV in the bedroom, sleep, and routine schedule) that were positively associated with becoming overweight and obese. The current version consists of 20 questions assessing the ten constructs mentioned above. The score ranges from 20 to 80 with lower scores indicating an adverse, obesogenic family environment. Two papers have shown its predictive validity of assessing the prevalence of overweight at baseline [17] and 1-year change in BMI after accounting for initial BMI, parent BMI, and other demographic variables [18].

### 3.3. Secondary Outcomes

#### 3.3.1. Physical Characteristics

Stature is measured without shoes to the nearest 0.1 cm using a ShorrBoard stadiometer (ShorrProduction, Olney, MD). Body mass is measured to the nearest 0.1 kg and body fatness (%BF) is estimated using a foot-to-foot bioelectric impedance scale (Tanita BC-534, Tokyo, Japan, and US Service Center Arlington Heights, IL). Body mass index (weight in kg/height in m^2^) is then calculated from measured stature and body mass. Age- and sex-specific percentiles for height, weight, and BMI are determined using the CDC SAS growth software (http://www.cdc.gov/nccdphp/dnpao/growthcharts/resources/sas.htm). Waist circumference is measured as a proxy for abdominal or visceral adiposity using a Gullick tape to the nearest 0.1 cm at the superior border of the iliac crest.

### 3.4. Feedback

Upon the completion of a session, the site coordinator is issued a report on the composite results, that is, attrition rate and mean values from pre- and postassessments and so forth. Patients are mailed a copy of a report card and cover letter that includes baseline and follow-up values for sleep, screen time, physical activity, fruit and vegetable consumption, low-fat dairy consumption, and the FNPA. In addition, the recommendation for each item is also indicated along with a brief statement about the behavior (e.g., children should have less than 2 hours of screen time per day). A separate report is sent to the referring physician and also includes educational insight into the key lifestyle behaviors and obesity assessment. In addition, the physician is reminded of the recommended visit schedule for stage 2 obesity treatment (monthly).

## 4. Costs

FitKids360 is offered free of charge to those who qualify. The program is of relatively low cost—about $196 per child at a participation rate of 20 children per seven-week session. This calculation is based on the budget shown in Table 4. However, given the interest in pediatric obesity within some communities and by some individuals, costs may be lower as some institutions will allow free facility rental or donate items (snacks, prizes, etc.). In addition, this does not include the cost (salary and benefits) for a program coordinator if multiple classes are run in a single community.

## 5. Meetings and Training Program

### 5.1. Meetings

To increase engagement amongst those interested in FitKids360, quarterly workgroup meetings are held and attended by key personnel, staff from existing FitKids360 sites, and any other interested persons or parties. These 1-2 hour meetings include presentations and discussion on topics such as class updates, recent results, funding opportunities, procedural changes, brainstorming sessions, and alerts to related opportunities in the community (i.e., 5 K walks, summer camps, etc.). As needed, subcommittees are formed and meet periodically. The entire program is overseen by a steering committee comprised of six individuals including two pediatricians, an exercise physiologist, a registered dietician, a researcher, and the program coordinator. The steering committee meets monthly and addresses all pertinent issues to the FitKids360 program.

### 5.2. Training Program

All “teams” interested in conducting a FitKids360 class are required to participate in our 1-day training program. Training sessions are held on a quarterly basis, depending on interest, and are provided by the program coordinator and several members of the FitKids360 steering committee, including the dietician, exercise physiologist, and researcher. The training session begins with an overview and introduction of the FitKids360 program (60 minutes). Given the ethnic and socioeconomic variation in our patients and their perceptions and beliefs about a healthy lifestyle, nutrition, food preparation, and so forth, we provide training on cultural competency and sensitivity (60 minutes) led by a community health educator with experience on this topic. Goal setting and motivational interviewing are taught and time is allowed to practice motivational interviewing techniques (50 minutes). Following the lunch break, an overview of assessments and measurements is provided (60 minutes), and then breakout groups are formed based on area of expertise (site coordinators and physicians, registered dieticians, behavioral health specialists, and exercise specialists). This portion of the training lasts about 2 hours to allow an in-depth training on each week of the curriculum. While lesson plans have been developed to engage the entire family, there are cognitive and developmental differences inherent in the wide age range of our patients. Therefore, these breakout sessions include training for adapting the curriculum to different age groups and provide examples of accommodations for younger and older participants. In addition, the FitKids360 “Buddy program” (described previously) also plays a major role in providing individualized support to both older and younger students. The training concludes with a question and answer session (30–45 minutes).

## 6. Outcomes

### 6.1. Data Analysis

Over the past 4 years a total of 33 FitKids360 classes have been administered at over dozen sites throughout the state of Michigan. Patient outcomes from these classes are presented below. Specifically, baseline characteristics were calculated as means ± standard deviations (SD), and changes in primary and secondary outcomes over the 7-week program were evaluated using within-subjects repeated measures analysis of variance. Before analyses, data were screened for outliers as identified by values >3 SD from the mean. All analyses were performed using PC-SAS (version 9.3) and alpha was set at *P* < 0.05.

### 6.2. Results

A total of 418 overweight or obese (BMI ≥ 85th centile) patients aged 5–16 years old were enrolled in one of 33 FitKids360 classes. 258 patients completed the program and the follow-up evaluation (62% retention). Patients were 10.5 ± 2.9 years old at baseline and 59% were female. Upon enrollment, 365 patients (87.3%) were obese (BMI ≥ 95th centile) and 173 (41.4%) of the total sample were severely obese (BMI ≥ 99th centile).

Mean changes in health behaviors are presented in Table 5. At baseline, 41% of patients reported meeting current physical activity recommendations (MVPA ≥ 60 min/d). Overall, patients increased MPVA by 14 min/d after completing FitKids360 (*P* = 0.019). Among those not meeting recommended activity levels at baseline, MVPA increased by 31 min/d and 56% reported MVPA levels sufficient to meet the 60 min/d guideline by follow-up. On average, patients reduced screen time by 44 min/d (0.7 hr/d) (*P* < 0.001) due to significant decreases in TV viewing (*P* < 0.001) and video game playing (*P* = 0.027). Among the 70% reporting >2 hr/d of TV viewing at baseline, 42% reduced to <2 hr/d after completing FitKids360.

Significant changes in reported nutrition behaviors included a reduction in the frequency of consumed sweets/desserts (*P* < 0.001) and sugar-sweetened soft drinks (*P* = 0.016) and an increase in the frequency of consumed whole grains (*P* < 0.001) and fruits and vegetables (*P* = 0.017) (Table 4). In addition, FNPA scores increased by 5.4 points (9%) in the overall sample (*P* < 0.001), and scores improved by 8.5 points (16%) among families identified as having high-risk family environments and behaviors according to baseline FNPA levels [17].

Anthropometric changes are displayed in Table 6. On average, patients increased both height (*P* < 0.001) and weight (*P* = 0.030) over the 7-week program resulting in a small but significant reduction in BMI (*P* = 0.011). Similarly, a significant decrease was measured in age- and sex-adjusted BMI *z*-scores (*P* < 0.001). Waist circumference and body composition estimates remained relatively unchanged after the program.

## 7. Discussion

Based on the results, it appears that FitKids360 may be an effective program for initiating positive health behavior changes and supporting weight management efforts, at least in the short term. Patients who completed FitKids360 made meaningful improvements to their physical activity and sedentary behaviors and reported significant progress in their nutrition habits, including consuming whole grains, fruits, and vegetables more frequently and sweets/desserts and sugar-sweetened soft drinks less frequently.

Perhaps most significantly, participants (and their families) improved overall health behaviors (i.e., household rules and family structure regarding diet, physical activity, sedentary time, and sleep) as indicated by the FNPA survey. Ihmels et al. have shown that youth in the lowest tertile for FNPA scores had 1.7 times higher odds of being overweight or obese (BMI ≥ 85th percentile) when compared to youth in the highest tertile [17]. In the current study, 50% of patients had baseline FNPA scores within this lowest tertile range, but 69% of these “high-risk” patients improved FNPA scores sufficiently to move them into the middle or upper tertiles after completing FitKids360.

Due to the short duration of the FitKids360 curriculum, substantial changes in body composition were not expected. Yet, patients who completed the program did experience small, but significant improvements in both BMI and BMI *z*-scores over the 7-week program. This is a notable result since typical BMI trajectories for growing children in this age range are positive. Future research is needed to assess the long-term sustainability and health impact of the health behavior and anthropometric changes that took place during FitKids360 classes.

One limitation of this study is the lack of a control/comparison group, which introduces several potential threats to internal validity, including confounding, regression toward the mean, and mortality. In addition, several of the lifestyle behaviors evaluated in this study were self- or parent-reported, which introduces potential social desirability bias. However, the self-report tools used in FitKids360 consist of surveys and questions which have been previously validated. In addition, the results presented here are fairly robust, as they include a relatively large sample from over 30 different FitKids360 interventions. Moreover, the FitKids360 program was designed as an evidence-based, yet feasible and practical pediatric weight management program rather than a randomized clinical trial. To this end, the data presented here provide a “real-world” indication of short-term outcomes after participating in the FitKids360 program.

The FitKids360 team is currently working to develop a standard follow-up procedure to allow long-term tracking of patients. We have initiated the process by sending a report to the patients' physicians asking them to continue long-term follow-up. Based on the expert recommendations [2], monthly office visits for 3–6 months are probably most appropriate at this level.

## 8. Summary

In summary, we have described the program design and intervention and measurement components of a low-cost stage 2 pediatric obesity program called FitKids360, which has been implemented in several locations throughout Michigan and has now expanded outside of the state as well. In addition, we have reported outcomes on over 250 youth who have completed the FitKids360 program, including improvements in physical activity, nutrition, and sedentary behaviors, as well as BMI. We hope that this report will be useful to researchers, public health professionals, and medical professionals seeking to develop similar stage 2 pediatric obesity programs.

## Figures and Tables

**Table 1:** Roles and responsibilities of each staff member involved in FitKids360.

Title	Role and responsibilities
Program director	This is generally a pediatrician or health care provider (e.g., physician assistant). Select and train all staff, arrange parent orientation, deliver orientation lecture, arrange class location, purchase incentive prizes and weekly equipment for children, review all child applications for appropriateness, arrange necessary technology for each class, prepare all handouts and slides needed weekly, plan weekly schedule of speakers and activities, confirm speakers' schedules and class time and location weekly, review data collection each week, arrange one-month, six-month, and one-year follow-ups, obtain children's measurements at follow-up, administer follow-up parent and children's surveys and final evaluations, transmit all data to research team, and coordinate data analysis.

Administrator	Prepare all forms (patient sign-up, physician referral/approval, informational flyer, and child and parent sign-in sheets), track points, fill out nametags, greet families, transport prizes and supplies, make weekly reminder phone calls, and make follow-up calls for missed classes.

Registered dietitian	On a weekly basis, prepare and deliver lectures, plan and run nutrition activities, purchase and prepare snacks, arrange grocery store tour, and run a cooking demonstration.

Behavioral health provider	This individual could be a developmental/behavioral pediatrician, pediatric psychologist, or medical social worker. Prepare and deliver orientation lecture, perform a motivational assessment of the parents, prepare and deliver weekly behavioral lecture and activities, and purchase supplies for weekly behavior activities as needed.

Exercise specialist	This individual could be physical therapist or recreational therapist or someone with a B.S. degree in exercise science or related field. Deliver weekly lectures regarding physical activity and screen time (sedentary behavior) and help plan weekly physical activities.

Personal trainer/fitness instructor	This individual could also be the exercise specialist. Plan and run weekly physical activities and oversee content of physical activities, including music, level of exertion, age-appropriateness, and appropriate teaching style for working with overweight children and adults.

Volunteers	Help with set-up, help prepare snacks and water, participate in physical activities and other group activities, collect paperwork, and socialize with kids and parents.

Data collectors	Measure orientation and administer surveys to parents and youth, train kids and parents to use pedometers and record their data, measure kids at final class and administer surveys to parents and kids again, and analyze all data at end of program.

**Table 2:** The FitKids360 curriculum. ^∗^ = patients and parent(s), ^∗∗^ = parents only, and ^∗∗∗^ = patients only. Each class begins with 10 minutes of group discussion regarding goals. Each class ends with 5 minutes to set and write new goals.

Week	Behavior	Nutrition	Exercise/screen time
1	Family goals Identify family support groups (30 min)^∗^	“Healthy counts” (8-7-6-5-4-3-2-1-0) Food guide pyramid (30 min)^∗^	FITT (15 min)^∗^ Group exercise (30 min)^∗^

2	Emotions (60 min)^∗∗∗^	Label reading (40 min)^∗∗^	Activity circle (15 min)^∗^ Group exercise (30 min)^∗^

3	Bullying (20 min)^∗^ Bullying (10 min)^∗∗∗^	Portion size (20 min)^∗^ Drinks (10 min)^∗^	Role modeling (15 min)^∗∗^ Game-chore game (10 min)^∗∗∗^ Group exercise (35 min)^∗^

4	Self-esteem (40 min)^∗∗^	Meal planning Healthy snacking (40 min)^∗∗^ Make healthy snack (30 min)^∗∗∗^	Screen time (20 min)^∗^ Game-commercial break activities (15 min)^∗∗∗^ Group exercise (35 min)^∗∗∗^

5	Communication (15 min)^∗∗^ Stress (15 min)^∗∗^	Eating out school lunches (30 min)^∗^	Exercise discussion (15 min)^∗^ Game^∗∗∗^ Group exercise (30 min)^∗^

6	Discipline/structure (30 min)^∗∗^ Share successes/award incentives (30 min)^∗^	Jeopardy game (nutrition and exercise) (30 min)^∗^	Game^∗∗∗^ Final biometric assessment (30 minutes)^∗∗∗^ Group exercise (20 min)^∗^

**Table 3:** Incentive point system for FitKids360.

Category	Points
Attendance	Earn 10 points for coming to class
Turn in diet log	Earn 10 points for turning in diet log
Turn in exercise log	Earn 10 points for turning in exercise log
Turn in screen time log	Earn 10 points for turning in screen time log
Good behavior	Earn 30 points per class

**Table 4:** Approximate expenses to conduct a FitKids360 class.

Facility rental	$400/class	$400
Instructor stipends	$50/person × 3 instructors × 7 sessions	$1050
Class materials	$7/participant × 20 patients	$140
Incentives and prizes	$600/class	$600
Nutritious snacks	$40/class × 7 sessions	$280
Transportation	$150/class × 7 sessions	$1050
3-month follow-up	$400	$400
Total		**$3920**
		$196 per patient

**Table 5:** Health behavior changes among youth who completed FitKids360.

	Baseline		Follow-up		Change		*P* value
	Mean	(SD)	Mean	(SD)	Mean	(SD)	
MVPA (min/d)	86.4	72.3	100.3	74.5	13.9	71.9	0.019
Screen time (hr/d)	4.7	2.8	4.0	3.2	−0.7	2.9	<0.001
Television (hr/d)	2.9	1.7	2.3	1.7	−0.6	1.7	<0.001
Videogames (hr/d)	0.9	1.0	0.7	1.1	−0.1	1.0	0.027
Computers (hr/d)	1.0	1.2	0.8	1.2	−0.1	1.4	0.171
FNPA (total score)	57.3	7.3	62.7	7.2	5.4	6.9	<0.001
Whole grain (freq/d)	1.5	1.4	2.2	1.6	0.7	1.8	<0.001
Dairy (freq/d)	2.0	1.4	2.1	1.5	0.1	1.5	0.413
Sweets/desserts (freq/d)	1.0	1.0	0.7	0.7	−0.3	1.1	<0.001
Sugar soft drinks (freq/d)	0.7	1.1	0.5	0.8	−0.2	1.1	0.016
Fruit juice (freq/d)	1.1	1.2	0.9	1.1	−0.1	1.4	0.204
Fruits + veg. (freq/d)	3.5	2.3	4.1	2.4	0.5	2.8	0.017

FNPA = family nutrition and physical activity survey; MVPA = moderate-to-vigorous physical activity; SD = standard deviation; veg. = vegetables.

Discrepancies in change scores are due to rounding error.

**Table 6:** Anthropometry changes among youth who completed FitKids360.

	Baseline		Follow-up		Change		*P* value
	Mean	(SD)	Mean	(SD)	Mean	(SD)	
Height (cm)	144.7	15.4	145.4	15.4	0.7	0.7	<0.001
Weight (kg)	61.2	24.0	61.5	24.1	0.3	2.2	0.030
BMI (kg/m^2^)	28.1	6.2	28.0	6.2	−0.2	1.0	0.011
BMI *z*-score	2.19	0.44	2.16	0.44	−0.03	0.10	<0.001
Percent body fat (%)	37.4	7.9	37.5	7.9	0.0	2.1	0.811
Waist circumference (cm)	87.7	16.4	87.9	16.3	0.2	5.1	0.477

Discrepancies in change scores are due to rounding error.

SD = standard deviation.
